# Endocytic Protein Defects in the Neural Crest Cell Lineage and Its Pathway Are Associated with Congenital Heart Defects

**DOI:** 10.3390/ijms22168816

**Published:** 2021-08-16

**Authors:** Angelo B. Arrigo, Jiuann-Huey Ivy Lin

**Affiliations:** 1 Department of Developmental Biology, University of Pittsburgh, Pittsburgh, PA 15224, USA; vns7qh@virginia.edu; 2 Department of Critical Care Medicine, University of Pittsburgh, Pittsburgh, PA 15224, USA

**Keywords:** neural crest cell, LRP1, LRP2, endocytic vesicle trafficking protein, congenital heart defect, double outlet right ventricle, common arterial trunk

## Abstract

Endocytic trafficking is an under-appreciated pathway in cardiac development. Several genes related to endocytic trafficking have been uncovered in a mutagenic ENU screen, in which mutations led to congenital heart defects (CHDs). In this article, we review the relationship between these genes (including *LRP1* and *LRP2*) and cardiac neural crest cells (CNCCs) during cardiac development. Mice with an ENU-induced *Lrp1* mutation exhibit a spectrum of CHDs. Conditional deletion using a floxed *Lrp1* allele with different Cre drivers showed that targeting neural crest cells with Wnt1-Cre expression replicated the full cardiac phenotypes of the ENU-induced *Lrp1* mutation. In addition, LRP1 function in CNCCs is required for normal OFT lengthening and survival/expansion of the cushion mesenchyme, with other cell lineages along the NCC migratory path playing an additional role. Mice with an ENU-induced and targeted *Lrp2* mutation demonstrated the cardiac phenotype of common arterial trunk (CAT). Although there is no impact on CNCCs in *Lrp2* mutants, the loss of LRP2 results in the depletion of sonic hedgehog (SHH)-dependent cells in the second heart field. SHH is known to be crucial for CNCC survival and proliferation, which suggests LRP2 has a non-autonomous role in CNCCs. In this article, other endocytic trafficking proteins that are associated with CHDs that may play roles in the NCC pathway during development, such as AP1B1, AP2B1, FUZ, MYH10, and HECTD1, are reviewed.

## 1. Introduction

During heart development, cardiogenic precursors form a cardiac crescent at around embryonic day (E) 7.5 in mice and at around day 15 of gestational age in humans. The cardiac crescent subsequently forms a linear beating heart tube by converging along the ventral midline of the embryo at E8.5 in mice and around day 21 of gestational age in humans. This linear heart tube is composed of a myocardial layer, an endocardial layer, and an extracellular matrix between these two layers. Although still primitive, the linear heart tube is segmentally patterned along the anterior–posterior axis into the future aortic sac, outflow tract, pulmonary ventricle, systemic ventricle, and atria. This linear heart tube undergoes rightward looping in all vertebrates. Cardiac chambers balloon out from the outer curvature of the lopped heart tube in a segmental fashion [1]. A single OFT then forms that subsequently divides to the aorta and pulmonary arteries. During this developmental window, endocardial mesenchymal transition (EMT) occurs, generating cardiac cushions that later remodel to form the semilunar and atrioventricular valves [2,3]. Neural crest cells are a transient, heterogenous, migratory, and multipotent cell population that originates from the dorsal neural tube in developing embryos [4]. Ablation of a subpopulation of neural crest cells resulted in a common arterial trunk (CAT) in a study by Dr. Kirby and collaborators [5]. Since then, this subregion of neural crest cells has been called the “cardiac neural crest” because this specific subpopulation of NCCs provides mesenchymal cells to the heart and the great arteries [5]. CNCCs originate from the neural folds of the developing hindbrain between the otic placode and the fourth somite, go through the epithelial–mesenchymal transition process, and then migrate to and populate specific areas of the embryonic heart [6,7]. The neural crest cell-derived mesenchymal cells are derived from the ectoderm and are referred to as “ectomesenchyme”. These cells are intrinsically different from “normal” mesenchymal cells that are derived from the mesoderm [8]. CNCCs migrate to the developing pharyngeal arches and outflow tract cushions and contribute to the smooth musculature of the tunica media of the ascending aorta and aortic arch, its major branches arch and provide a subpopulation of connective tissue cells to the semi-lunar valves [7,9]. CNCCs also participate in the development of the endocardial cushion [10], interventricular septum, coronary arteries, and the parasympathetic innervation of the heart [11,12,13,14]. Proper connection of the great arteries to their ventricles involves rotation of the developing great arteries and sandwiches the aorta between the atrioventricular valves [15]. CNCCs grow from the distal to proximal embryonic outflow tract and merge with other cell lineages such as the second heart field cells to form a septum [16]. This septum divides the single embryonic outflow tract into the aorta and main pulmonary artery [5]. Without this separation, the resultant congenital heart defect (CHD) involves a common arterial trunk [5]. Without appropriate and complete rotation, the resultant CHD is double outlet right ventricle (DORV) [17]. In this review, we will address the association of endocytic vesicle trafficking proteins with neural crest cells, in which mutations result in congenital heart defects.

Congenital heart diseases are the most common congenital defects and are the leading cause of neonatal death [18,19]. Despite a strong indication of a genetic contribution to CHDs, the developmental processes and genetic etiology are poorly understood. Given the difficulties with human genetic analysis, Dr. Lo and collaborators used a large-scale forward genetic screen of chemically mutagenized mice with ethyl-nitrosourea (ENU) to uncover the genetic etiology of CHDs [20]. This method enables an understanding of embryonic patterning by investigating phenotypes without a prior assumption of the genes or pathways that may be relevant [21]. Since mutant alleles that are induced by ENU often have relatively selective effects on protein function, they provide valuable information about the function of different protein domains. An unexpected role for the endocytic pathway in CHD pathogenesis was revealed by this large-scale mouse forward genetic screen [20]. Furthermore, a mouse model of a point mutation that mimics human disease provides an opportunity to understand the pleiotropy of human congenital heart defects and the mechanisms by which a single genetic alternation can result in a spectrum of cardiac phenotypes.

Endocytosis plays an important role in modulating cell signaling by regulating the internalization, recycling, and degradation of receptor–ligand complexes. Endocytosis regulates the trafficking of the internalized endocytic vesicles to different endosomal compartments, with some destined for fusion with lysosomes and degradation, while others are recycled to the surface by recycling endosomes [22,23].

## 2. LRP1 (Low Density Lipoprotein Receptor-Related Protein 1)

### 2.1. Lrp1 Mutation Is Associated with DORV and AVSD

Among the mutations involved in the endocytic trafficking proteins, a missense mutation in low-density lipoprotein receptor-related protein 1 (*Lrp1*), a member of the multifunctional low-density lipoprotein (LDL) receptor-related protein (LRP) family [24,25,26], was noted to result in CHDs [20,27]. LRP is the main receptor for the uptake of lipoproteins in vertebrate cells [26]. LRP1 is expressed as a 600 kDa precursor that is cleaved by furin, resulting in a 515 kDa extracellular ligand-binding α chain and a noncovalently bound 85 kDa membrane-bound cytoplasmic β-chain [24]. We have identified a mutant mouse line, 1554 (MGI 96828), that results in a missense (C4232R) mutation in the region encoding the epidermal growth factor (EGF) repeat domain of LRP1. The α-chain of LRP1 contains various repeated motifs and the β-chain contains the C4232R mutation and two cytoplasmic NPxY sequences that serve as signals for endocytosis [24,25]. By using different endocytic trafficking markers and Western blot, the C4232R mutant protein had a substantial reduction in the 85 kDa LRP1 β-chain expression secondary to altering endocytic trafficking with endoplasmic reticulum retention of the mutant LRP1^C4232R^ protein [27]. LRP1 was originally described to mediate endocytosis of lipoproteins, but it is now also appreciated that LRP1 can bind dozens of other ligands. This includes many different transmembrane receptors, with their activity and downstream intracellular signaling modulated by LRP1 regulation of their internalization, sorting to different endosomal compartments, their recycling, and their degradation [24,25,28].

**Figure 1:**
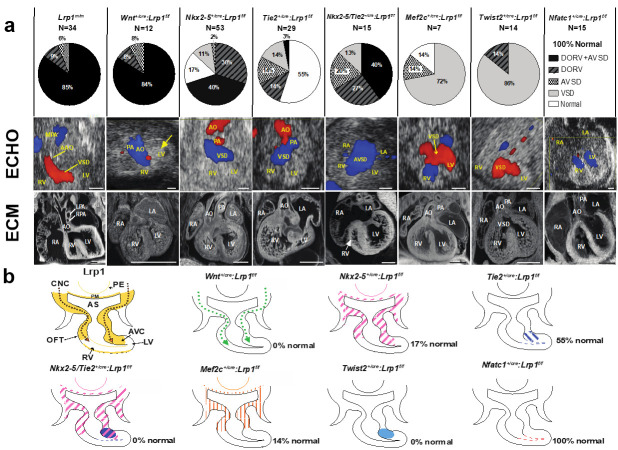
Conditional Cre deletion of the floxed *Lrp1* allele. Conditional *Lrp1* knockout in different cell lineages during cardiac development was carried out using different Cre drivers. (**a**) Pie charts illustrating the percentages of different cardiac phenotypes and *Lrp1^m/m^* cardiac phenotypes. (**b**) *Lrp1* expression is illustrated in yellow in developing heart around E10.5–E11.5. Targeting *Lrp1* deletion in neural crest cells using *Wnt1-Cre* (*Wnt1^+/Cre^*: *Lrp1^f/f^*) recapitulates the cardiac phenotypes of *Lrp1^m/m^* mutants, which is illustrated in dotted green arrows. The ablation of LRP1 expression using *Nkx2-5-Cre* (Nkx2-5*^+/Cre^*: *Lrp1^f/f^*) is illustrated in pink hash, specifically in the PM, PE, AHF, and AV canal cushion. The ablation of LRP1 expression using *Tie2-Cre* (*Tie2^+/Cre^*: *Lrp1^f/f^*) is illustrated in blue hash and blue dots. The ablation of *Lrp1* in double knockout of *Nkx2-5-Cre* and *Tie2-Cre* (Nkx2-5*^+/Cre^ Tie2^+/Cre^*: *Lrp1^f/f^*) is illustrated in pink and purple hash. The combined deletion of *Lrp1* mediated by *Tie2-Cre* and *Nkx2-5-Cre* together demonstrated the high penetrance of the DORV/AVSD phenotype and increased the penetrance of AVSD. The ablation of *Lrp1* using *Mef2c-AHF-Cre* (*Mef2c-AHF^+/cre^*: *Lrp1^f/f^*) is illustrated in orange hash. The ablation of *Lrp1* using *Twist2-Cre* (*Twist2^+/cre^*: *Lrp1^f/f^*) is illustrated in navy blue. There is no cardiac phenotype of *Nfatc^+/cre^*: *Lrp1^f/f^*; the ablation of LRP1 expression is expressed in red. AHF: anterior heart field; AO: aorta; AS: aortic sac; AVC: atrioventricular canal; AVSD: atrioventricular septal defect; CNCC: cardiac neural crest cells; DORV: double outlet right ventricle; LA: left atrium; LV: left ventricle, OFT: outflow tract; PA: pulmonary artery; PE: pharyngeal endoderm; PM: pharyngeal mesoderm; RA: right atrium; RV: right ventricle; VSD: ventricular septum defect. Scale bars: 0.5 mm (modified from reference [27] Figure 3, with additional data added).

Mice harboring the *Lrp1*^C4232R^ mutation exhibit homozygous lethality (*Lrp1^m/m^*) at E14.5–E15.5, with rare stillborn pups [27]. Cardiac assessments using fetal echocardiography and episcopic confocal microscopy (ECM) demonstrated that all *Lrp1^m/m^* mutants have congenital heart defects. The majority (85%) had the outflow tract malalignment defect of double outlet right ventricle (DORV) and an atrioventricular septal defect (AVSD) [27]; 9% had isolated DORV and 6% had isolated AVSD (Figure 1). A representative fetal echocardiogram and ECM demonstrated that the *Lrp1^m/m^* mutant showed a flow across the ventricular septum (ventricular septal defect, VSD) and side-by-side great vessels, with the aorta positioned to the right of the pulmonary artery (Figure 1).

### 2.2. Lineage-Specific Roles of Lrp1 in Cardiac Development

*LRP1* is expressed in multiple cell lineages that are crucial during heart development, including neural crest, first heart field, second heart field, and mesenchymal cells of the developing endocardial cushion [27]. A floxed *Lrp1 (Lrp1^f/f^)* allele [29] with different Cre drivers to orchestrate conditional *Lrp1* deletion in cell lineages associated with cardiac development was generated to investigate the pathomechanisms of DORV and AVSD. The Cre drivers used included Wnt1 (neural crest cell) [30,31], Mef2c (second heart field/anterior heart filed-Cre targeting cells) [32], Nkx2-5 (first heart field, second heart field, pharyngeal endoderm and pharyngeal mesoderm), Tie2 (endothelium/endocardium, and mesenchymal cells of atrioventricular cushion) [33], Nfatc-1 (endocardium) [34], and Twist-2 (mesenchymal cells in the endocardial cushion) [35,36] (Figure 1). *Wnt1^+/cre^*: *Lrp1^f/f^* mutant embryos did not survive past E14.5. Significantly, 100% of the Wnt*1^+/cre^*: *Lrp1^f/f^* embryos exhibited CHD: 83% with DORV and AVSD, 8% with DORV, and 8% with AVSD. This replicates the same CHD phenotype with similar penetrance to that seen in the *Lrp1^m/m^* mutant [27] (Figure 1). These findings suggest that CHD in the *Lrp1^m/m^* mutant may arise from a cell autonomous requirement for *Lrp1* in CNCC. An analysis of 53 *Nkx2-5^+/cre^*: *Lrp1^f/f^* mutant embryos to investigate the requirement for *Lrp1* in myocardial cells from the first and second heart field [37] resulted in 83% of mutants having CHD. This included AVSD and DORV, isolated DORV, membranous VSD, and unbalanced AVSD with a dominant left ventricle (Figure 1). Using the *Tie2-Cre* driver to target *Lrp1* deletion in the endothelial/endocardial lineage cells in the outflow tract, and the endocardial cushion mesenchyme [33], we observed that 45% of the 29 Cre-deleted embryos (*Tie2^+/cre^*: *Lrp1^f/f^*) had cardiac defects (Figure 1). Because *Lrp1* is not expressed in endothelial/endocardial cells but is highly expressed in the cushion mesenchyme, this suggests that the CHD resulting from *Tie2-Cre* deletion [33] may reflect a requirement for *Lrp1* function in cushion mesenchyme. Further assessment of the *Lrp1* deletion using the mesenchymal-specific *Twist2-Cre* [35,36] showed that the mutant embryos with *Twist2-Cre* [35,36] deletion (*Twist2^+/cre^: Lrp1^f/f^*) had septal defects with membranous VSD or isolated DORV. In contrast, all embryos generated with endocardial specific *Nfatc1-Cre* [34] deletion (Nfatc1*^+/cre^*: *Lrp1^f/f^*) had normal cardiovascular anatomy, suggesting that the cushion mesenchymal cells targeted by *Tie2-Cre* may not derive from an Nfatc1-positive endocardium [27] (Figure 1). When *Lrp1* was knocked out using the *Nkx2-5-Cre* [37] and *Tie2-Cre* [33] drivers simultaneously to investigate if *Lrp1* in myocardial cells and in non-endocardium-derived cushion mesenchyme contributed synergistically to heart development, this resulted in 100% of the *Tie2^+/Cre^/Nkx2-5^+/Cre^*: *Lrp1^f/f^* mutant embryos exhibiting a CHD. (Figure 1). A 13-fold increase in the incidence of DORV with AVSD—40% with *Tie2^+/Cre^/Nkx2-5^+/Cre^*: *Lrp1^f/f^* double Cre deletion vs. 3% with *Tie2^+/Cre^*: *Lrp1^f/f^*—was observed. This observation suggested that *Lrp1* in myocardial cells and in the non-endocardium-derived cushion mesenchyme is essential for supporting cardiac development [27]. These Cre drivers have broad expression domains encompassing embryonic tissues with *Lrp1* expression found along the CNCC migratory path, including the myocardium of the first/second heart field, pharyngeal endoderm, mesoderm, and cushion mesenchyme. Hence, *Lrp1* expression in these additional cell lineages may provide a signaling function that can ensure quantitative recruitment of neural crest cells to the heart. Consistent with this, *Nkx2-5^+/Cre^/Tie2^+/Cre^*: *Lrp1^f/f^* double Cre deletion exerts additive effects, yielding more severe phenotypes with higher disease penetrance than either Cre driver alone. Together, these data suggest *Lrp1* function is primarily required in the CNC lineage and is a requirement in the myocardial cells of the first and second heart field and in the cushion mesenchyme of non-endocardial origin as well.

### 2.3. LRP1 Is Required for Cardiac Neural Crest Cell Migration

Neural crest cells require LRP1 to migrate to the cardiac OFT properly. This is shown by the following observations. (1) There was a marked decrease in LacZ-expressing cells in the atrioventricular cushion and OFT cushion of E10.5 *Lrp1^m/m^* mutant embryos as compared to controls when using a *Wnt BAT-LacZ* reporter [38,39]. This was associated with hypo-cellularity, which is characterized by decreased abundance of mesenchymal cells in the endocardial and OFT cushions [27]. These results are consistent with reduced cell proliferation and increased apoptosis observed earlier in the E10.5 embryonic AVC and OFT cushions of *Lrp1^m/m^* mutants [27]. (2) Immunochemistry using AP2-α [31], a marker for neural crest cells, and LRP1 in E10.5 embryos demonstrated that *Lrp1^m/m^* mutants had significantly decreased expression of AP2-α and LRP1 in the developing OFT. (3) The conditional deletion of LRP1 in the neural crest lineage using a *Wnt1-Cre* [30,31] driver results in outflow tract defects (DORV) and septation anomalies (AVSD). (4) A Cre lineage tracing of neural crest cells using *Wnt1-Cre* [30,31] with a Rosa/LacZ reporter [40] demonstrated decreased expression of Wnt1/LacZ cells in the outflow tract of E10.5 embryos, which is consistent with decreased Wnt BAT-LacZ expression in the AV and OFT cushions of *Lrp1^m/m^* mutants [27]. This suggests that perturbation of WNT signaling in *Lrp1^m/m^* mutants is associated with the reduced expression of CNCCs.

The LRP1 C4232R mutated protein results in cell migration defects secondary to reduced focal adhesion turnover. CNCCs originate from the hindbrain neural fold and migrate into the pharyngeal arches and the developing heart. Given the importance of directional cell migration in the targeting of neural crest cells to the heart, the observation of a significant reduction in the rate of wound closure due to alterations in the speed of cell locomotion in the *Lrp1^C4232R^* mutant (*Lrp1^m/m^*) [27] suggested that a cell migration defect in *Lrp1^m/m^* is critical in causing cardiac phenotypes. LRP1 regulates the trafficking of integrins; integrins regulate focal adhesions and link the extracellular matrix to the actin cytoskeleton for cell migration and matrix adhesion [41]. Ablation of mesodermal integrin α5β1 resulted in arch anomalies and ventricular septal defect secondary to dysfunction in neural crest-derived cells [42]. *Lrp1^m/m^* mouse embryonic fibroblasts (MEFs) demonstrated a significantly reduced focal adhesion turnover rate, as studied using live cell imaging with transient transfection of a vinculin-green fluorescent protein (GFP) reporter [27]. These findings suggest a primary CNCC migration defect may drive the OFT malalignment and shortening OFT phenotypes in the *Lrp1^m/m^* mutant. A study of mice with *Nkx2-5-Cre*-mediated knockout of focal adhesion kinase yielded CHD comprising DORV or an overriding aorta [43]. As *Nkx2-5-Cre*-deletion of *Lrp1* also generated DORV, but with reduced penetrance compared to the *Wnt1^+/Cre^*: *Lrp1^f/f^*, this would suggest there is a secondary requirement for *Lrp1* in Nkx2.5 expression cells to orchestrate normal OFT lengthening.

### 2.4. LRP1 Is Essential for Cardiac Outflow Tract Development and Endocardial Cushion Maturation

More than 90% of *Lrp1^m/m^* (91%: 6% with AVSD, 85% with AVSD and DORV) mice and Wnt*1^+/cre^*: *Lrp1^f/f^* mutants (92%: 8% with AVSD, 84% with AVSD and DORV) had AVSD. LRP1 is expressed strongly in the mesenchyme cells of the developing endocardial cushion in the developing mouse. There are several lines of evidence demonstrating altered EMT in *Lrp1^m/m^* mutant mice. (1) The *Lrp1^m/m^* mutant heart revealed a single primitive, undivided atrioventricular (AV) cushion as compared to two distinct, well-separated AV cushions at E12.5. (2) Cardiac cushions at E10.5, including the OFT and AVC, are dysmorphic and hypocellular in *Lrp1^m/m^* mice. The development of cardiac cushions depends strongly on EMT for development [2]. (3) Defective EMT in the absence of LRP1 is also supported by decreased migration in isolated *Lrp1^m/m^* fibroblasts, as well as the explants from developing atrioventricular cushions [27]. These observations are consistent with prior reports linking LRP1 with fibroblast development and EMT. LRP1 activates ERK1/2 signaling to promote fibroblast proliferation, survival, and profibrotic transdifferentiation during inflammatory responses [44,45]. In the development of the heart valves, the transcription factor Yes-associated protein (Yap1) regulates heart cushion EMT [46]. There is a positive correlation between YAP1 and LRP1 expression [47]. Therefore, Yap1 may drive the transcription of LRP1 [46,47], likely making these cells more responsive to other pro-EMT signals. Given the close association between LRP1 and cell motility, one can expect that degrading the protein, or sequestering the protein in the endoplasmic reticulum, should inhibit EMT and lead to a defect in the formation of greater vessels and atrio-ventricular septa from epithelial/endothelial precursors. Our observation of defective EMT in the absence of LRP1 is also supported by experiments using isolated *Lrp1^m/m^* fibroblasts (MEFs). The defective *Lrp1^m/m^* fibroblast migration is caused by greater adhesiveness of focal adhesions as mentioned in the section of LRP1 and the developing OFT. Explanted *Lrp1^m/m^* E10.5 heart cushions migrate much more slowly into a gel matrix, indicating that EMT is significantly inhibited in this tissue.

These observations in the *Lrp1* mouse model suggest CNCCs impact cardiac development through regulation of OFT lengthening by providing migratory cues for recruiting second heart field cells and endothelial-derived cells to the distal OFT. Consistent with this, CNCC ablation in *Lrp1* leads to shortening of the OFT, with fewer myocardial cells added to the distal OFT [11]. In this *Lrp1* mouse model, for the first time we provided the observation that a deficiency in an endocytic trafficking protein in the CNCC can cause CHD due to the disruption of OFT lengthening and a perturbed OFT alignment, resulting in DORV. A defect in LRP1 in CNCC can cause abnormalities in expansion of AV cushion mesenchyme [10,27]—this cushion defect may cause AVSD.

**Figure 2:**
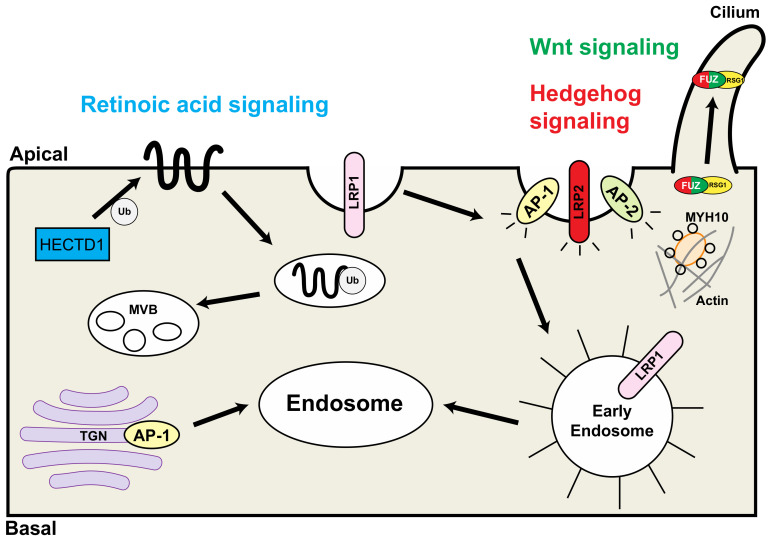
Schematic representation of the interaction of endocytic proteins that are associated with congenital heart defects. HECTD1 is involved in the retinoic acid pathway (blue); LRP2 and FUZ are involved in the hedgehog signaling pathway (red); FUZ is involved in the Wnt signaling pathway (green). TGN: trans-Golgi network; Ub: ubiquitin; MVB: multivesicular bodies; RSG1: RAB-like small GTPase 1. Modified from reference [20], Figure 2.

Other endocytic trafficking proteins are associated with congenital heart defects and may play essential roles in neural crest function during development (Table 1, Figure 2).

## 3. LRP2 (Low-Density Lipoprotein Receptor-Related Protein 2)

LRP2, also known as megalin, is also a member of the low-density lipoprotein (LDL) receptor-related protein (LRP) family. LRP2 is a glycoprotein with a large extracellular domain, (which is required for endocytosis), a single transmembrane domain, and a short carboxy-terminal cytoplasmic tail [26,48]. The extracellular domains are required for cell-surface ligand binding and ligand releases [26]. LRP2 binds diverse signaling molecules, including sonic hedgehog (SHH) [49,50,51], bone morphogenetic protein (BMP4) [49,50], and retinol binding protein (RBP) [51]. LRP2/megalin was identified as an autoantigen in glomerular podocytes [52]. The interaction of the LRP2/megalin autoantigen with autoantibodies causes Heymann nephritis [52]. Subsequently, a giant 600 kDa protein was cloned to be the major autoantigen in Heymann nephritis on the surface and was termed megalin (or Lrp2 or gp330) [53]. Although the role of LRP2 in renal pathophysiology received major attention initially, the phenotypic consequences of LRP2 deficiency first noted in mouse models and in humans were defects in brain development [50,52,54]. LRP2 is expressed in the neuroepithelium and loss of LRP2 in the knockout mouse model results in holoprosencephaly (fusion of the forebrain) [50] and buphthalmia (the overgrowth of the eye globe) [49]. Similar features are noted in patients with Donnai-–Barrow/facio-oculo-acoustio-renal syndrome, an autosomal recessive disorder caused by *LRP2* mutations [55,56,57].

Targeted [58,59] or ENU-induced *Lrp2* [20] mouse mutants were characterized by the cardiac phenotypes of CAT (single outflow tract without separating aorta and pulmonary artery) and DORV [58,59]. CAT is the result of the absence of outflow septation. LRP2 is expressed in the cardiac progenitor cells of the anterior second heart field, which is critical in the developing heart for elongation of the outflow tract and separation of the aorta and main pulmonary artery [59]. By using the sonic hedgehog (SHH) pathway reporter Gli1-LacZ and tamoxifen-induced Gli1 conditional knockout in LRP2, Christ et al. elegantly demonstrated that LRP2 regulates SHH-dependent progenitor cells in the anterior second heart field [59]. *Lrp2^−/−^* knockout mice demonstrated a failure to respond to SHH [50]. In addition, mutations in key SHH pathway genes, including ligands such as SHH and downstream signaling cascade member smoothened (SMO), cause CAT [60]. Therefore, the cardiac phenotypes of *Lrp2^−/−^* knockout mice mimic those of the key sonic hedgehog (SHH) genes knockouts. Furthermore, *LRP2* is known to act as an SHH-binding protein that controls cell trafficking of SHH/Patched 1 complex [50,59]. Using a series of tissue-specific conditional knockouts, Goddeeris et al. demonstrated that the SHH ligand is essential for CNCC to contribute to the outflow tract cushions. Furthermore, SHH is crucial for the second heart field cells to separate the developing outflow tract through mediating signaling to the cardiomyocytes [60]. Ablation of SHH in the Nkx2-5 Cre domain (*Nkx2-5^Cre/+: Shhf/−^*) resulted in the cardiac phenotype of CAT with reduction in the length of OFT and right ventricle at E10.5 [60]. Although LRP2 deficiency did not have an obvious effect on neural crest migration into the outflow tract [59], loss of LRP2 in knockout mice does have an impact in SHH, which is essential for CNCC migration [60]. Together, these data suggest LRP2 as a receptor in the myocardial cells derived from the second heart field [59] that interacts with the SHH ligand and is required during outflow tract formation [60].

## 4. AP1B1 (Adaptor Related Protein Complex 1 Subunit Beta 1)

AP1B1 is a clathrin-associated adaptor protein (AP) complex that plays a role in endocytic protein sorting in the Golgi network and endosomes [61]. AP1B1 plays an important role in somatodendritic sorting in neurons [62]. An ENU-induced mutation was uncovered with a T (thymine) to C (cytosine) substitution at coding nucleotide position 1094 in exon 9 of the cDNA (c.1094T>C, NM_007454). The homozygous mice that carry this V365A mutation are associated with laterality defects, including heterotaxy, dextrocardia, right arch, DORV, bilateral inferior vena cava, and septation defects in the heart including atrioventricular septal defects and ventricular septal defects [20]. The related craniofacial defects in these *Ap1b1^V365A/V365A^* mice, including cleft palate and micrognathia, imply its association with neural crest cell defects. Homozygous mutations in *AP1B1* were noted to be associated with mental retardation, neuropathy, hydrocephalus, microcephaly, and hypotonia in humans [61,63].

## 5. AP2B1 (Adaptor-Related Protein Complex 2 Beta1)

AP2B1 is an adaptin protein complex 2 subunit that mediates in clathrin-mediated endocytosis. AP2B1 and AP1B1 are members of adaptor protein complexes [62]. The mutation of a T to A (substitution at position 1343 of the cDNA (c.1343T>A, NM_027915) results in a change of a methionine residue to lysine at position 448 of the encoded protein (p.M448K). Mice that carry this homozygous mutation exhibit cardiac phenotypes of DORV, AVSD, and arch anomalies [20]. The extra-cardiac phenotypes of the *Ap2b1^M448K/M448K^* include micrognathia, cleft palate, and thymus hypoplasia reminiscent of neural crest anomalies. AP2B1 protein participates in pre- and post-synaptic endocytosis in neurons for both clathrin-dependent sorting and microtubule-dependent transport [62]. The cardiac phenotypes observed in *Ap2b1* mutants may be through its function in CNCC-derived mesenchymal cells in the developing heart.

## 6. Fuz (Fuzzy Planar Cell Polarity Protein)

The *FUZ* gene encodes a planar cell polarity effector protein, FUZ. *FUZ* is one of the four endocytic trafficking protein genes (*LRP2*, *FUZ*, *DCTN5,* and *MYH10*) that are related to the ciliome (a proteome of cilia) and congenital heart defects. *FUZ* is important for normal trafficking of retrograde intraflagellar transport (IFT) in cilia [64]. The cardiac phenotypes of the homozygous mutant embryos include pulmonary atresia, atrioventricular septal defect, and right aortic arch [20]. The molecular lesion is a T to A substitution at nucleotide +2 after coding nucleotide 387 (c.387+2T>A, NM_027376.3) in intron 4. This results in altering the splice donor site G (guanine)-GT to G-GA, which is assumed to be much less efficient. *Fuz*^Gt1(neo)^ mutant mice were generated using a gene-trapping technique demonstrated a single outflow tract, neural tube closure defects (exencephaly, encephaloceles), and craniofacial malformations [65]. FUZ knockout (*Fuz^−/−^*) mice exhibit severe craniofacial anomalies, including cleft palate, a hypoplastic mandible, and anophthalmia. These birth defects in *Fuz^−/−^* mice are associated with down-regulation of the hedgehog pathway and upregulation of the canonical WNT pathway [66]. Loss of *Fuz* in a knockout mouse model leads to disorganized neural crest cell migration and an increase in neural crest cells in the first branchia arch, resulting in maxillary hyperplasia. These craniofacial defects are rescued in *Fgf8* heterozygous mice (Fgf8^LacZ/+^) [67]. Mutations in the *FUZ* gene are noted to be associated with neural tube defects in human [68]. These data suggest that FUZ, an endocytic protein that functions in cilia, may play a significant role in neural crest cells, in which a disturbance leads to CHDs.

## 7. Myh10 (Myosin Heavy Chain 10)

*MYH10* encodes myosin heavy chain-10 and is also known as non-muscle myosin IIB. MYH10 is essential for clathrin-mediated endocytosis [69]. A point mutation was generated using ENU, which resulted in a T to C substitution at coding nucleotide 1054 in exon 10 of the cDNA (c.1054T>C, NM_175260). This results in a change of a serine residue to proline at position 352 of the encoded protein (p.S352P). Cardiac phenotypes of DORV, AVSD, interrupted arch, and ventricular noncompaction were observed in the homozygous mutants (*Myh-10^S352P/S352P^*) [20]. DORV and VSD are also observed in a homozygous knock-in mutant mouse line (B^R709C^/B^R709C^). Extracardiac anomalies, including cleft palate, body wall defects (ectopia cordis and omphalocele), hydrocephalus, and abnormal neural migration, were noted in homozygous mutants (B^R709C^/B^R709C^ and *B^−^/B^−^*) [70]. Furthermore, *Myh10* is one of the down-regulated genes in the homocysteine-responsive transcriptome in chicken neural crest explants [71].

## 8. HECTD1 (HECT Domain E3 Ubiquitin Protein Ligase 1)

*HECTD1* encodes a E6-AP C terminal (HECT)-domain containing E4 ubiquitin ligase. The homozygous *Hectd1* mutants are generated from an ENU-induced mutation with a molecular lesion of a T to A substitution at coding nucleotide 3264 in exon 22 of the cDNA (c.3264T>A, NM_144788). This changes a tyrosine residue to a translation stop at position 1088 of the encoded protein (p.Y1088*). The cardiac phenotypes of the homozygous mutants (Hectd1^Y1088*/Y1088*^) demonstrated ascending aorta hypoplasia, aortic atresia, dysplastic semilunar valves, and ventricular septal defects [20]. Another ENU-induced *Hectd1* mutation (*open mind, opm*) exhibits exencephaly in the homozygous *opm* mutants (*Hectd1^opm/opm^*) [72] and arch anomalies in the heterozygous mutants (*Hectd1^opm/+^*) [73]. The neural tube closure defects in *Hectd1 opm* mutants are not derived from the neural crest, but from the cephalic paraxial mesoderm [72]. However, the abnormal arch morphologies observed in *Hectd1^opm/+^* are related to the retinoic acid pathway, and the frequency of arch anomalies can be altered by manipulating the dose of vitamin A [73]. Retinoid signaling plays a role in cardiac outflow tract development by impacting the second heart field [74] and CNCC [75]. In retinoic acid-deficient, hypomorphic *Raldh2* (retinaldehyde dehydrogenase 2) mutant mice had phenotype of early embryonic lethality with an un-looped cardiac ventricle. Using retinoic acid to rescue *Raldh2^−/−^* mutants results in conotruncal defects, including DORV, CAT, and tetralogy of Fallot. In this mouse model, fewer and disorganized CNCCs were observed in the developing outflow tract (E11.5–E12.5) of retinoic acid-rescued *Raldh2^−/−^* mutants [75]. A genetically engineered knockout mouse model of a retinoic-acid-responsive enhancer, *Hoxa3*, demonstrated phenotypes of CNCC ablation [76]. These observations suggest HECTD1 may play a role in CNCCs through the retinoic pathway.

## 9. Conclusions

In conclusion, we demonstrated that an under-appreciated endocytic trafficking pathway defect is associated with congenital heart disease through an autonomous effect from the neural crest cell lineage and a non-autonomous effect of cell lineages along the neural crest cell migrating pathway. Most of the mutations in endocytic trafficking proteins are associated with craniofacial defects including cleft palate and micrognathia, suggesting that neural crest defects are associated with congenital heart disease and craniofacial anomalies.

## Figures and Tables

**Table 1:** Summary of discussed molecules with related phenotypes in mouse and human disease. Along with identifiers for each gene, the nucleotide change, protein change, mouse phenotype, related pathways, and human disease are listed, if applicable.

Gene	Mouse Line ID	MGI ID	Nucleotide Change	Protein Change	Mouse Phenotype	Related Pathways	Human Disease
Ap1b1	1660	5433323	c.T1094C	p.V365A	Heterotaxy, AVSD, DORV, right arch, micrognathia, craniofacial defects		
Ap2b2	2321	5552944	c.T1343A	p.M448K	DORV/Taussig-Bing, AVSD/VSD, aortic arch defects, craniofacial defects		Ataxia telangiectasia, cerebellar degeneration
Fuz	1273	5311392	c.387+2T>A	This changes splice donor site G-GT to G-GA which is assumed to be much less efficient	Pulmonary atresia, AVSD, MAPCA, right arch, TEF, craniofacial defects, diaphragmatic hernia, limb defects	Hedgehog signaling WNT signaling	Neural tube defects
Hectd1	327	5313700	c.T3264A	p.Y1088X	Aortic atresia/hypoplasia, dysplastic semilunar valve, VSD, exencephaly, neural tube defects	Retinoic acid signaling	
Lrp1	1554	5437079	c.T12694C	p.C4232R	DORV, AVSD, pulmonary stenosis, craniofacial defects		Alzheimer disease, Schizophrenia
Lrp2	1625	5489925	c.8456-3A>G	Y2204X	PTA, IAA, AVSD, craniofacial defects	Hedgehog signaling	Donnai-Barrow syndrome
Myh10	2437	5552947	c.T1054C	p.S352P	DORV/OA, exencephaly, micrognathia, hydronephrosis, body wall defects		

AVSD: atrioventricular septal defect, DORV: double outlet of right ventricle, IAA: interrupted aortic arch, MAPCA: Major aortopulmonary collateral arteries, OA: overriding of aorta, PTA: persistent truncus arteriosus, TEF: tracheoesophageal fistula, VSD: ventricular septal defect.

## Data Availability

Not applicable.
